# Radiomic and clinical predictors of epidermal growth factor receptor mutation in stage IA non-small cell lung cancer

**DOI:** 10.7717/peerj.21579

**Published:** 2026-08-03

**Authors:** Wanxian Guan, Jiaqing Chen, Xueqing Zeng, Yulan Chen, Baoyi Liu, Pengaolong Xin, Zhizhen Huang, Woqing Chen, Yan Zheng, Jiarong Chen, Liangliang Ren, Dong Ren, Xin Zhang, Yanming Huang, Shengming Liu

**Affiliations:** 1 Department of Respiratory and Critical Care Medicine, The First Affiliated Hospital of Jinan University, Guangzhou, Guangdong, China; 2 Department of Respiratory and Critical Care Medicine, Jiangmen Central Hospital, Jiangmen, Guangdong, China; 3 Jiangmen Key Laboratory of Precision and Clinical Translation Medicine, Clinical Experimental Center, Jiangmen Engineering Technology Research Center of Clinical Biobank and Translational Research, Jiangmen Central Hospital, Jiangmen, Guangdong, China; 4 School of Electronics and Information Engineering, Wuyi University, Jiangmen, Guangdong, China; 5 Department of Research and Development, Guangdong Research Institute of Genetic Diagnostic and Engineering Technologies for Thalassemia, Hybribio Limited, Guangzhou, Guangdong, China; 6 Department of Oncology, Jiangmen Central Hospital, Jiangmen, Guangdong, China; 7 Department of Pathology, University of California Irvine Medical Center, Orange County, CA, United States

**Keywords:** Non-small cell lung cancer (NSCLC), Epidermal growth factor receptor (EGFR), Machine learning, CT features, Clinical features

## Abstract

**Background:**

Epidermal growth factor receptor (EGFR) mutation status plays a critical role in guiding targeted therapy for non-small cell lung cancer (NSCLC). However, molecular testing in patients with stage IA NSCLC may be limited by insufficient tissue availability, procedural invasiveness, and resource constraints. Therefore, developing a non-invasive approach for EGFR mutation prediction is of substantial clinical interest. This study aimed to develop a computed tomography (CT) radiomics based model integrating clinical variables for non-invasive prediction of EGFR mutation status in stage IA NSCLC patients.

**Methods:**

A total of 375 patients with stage IA NSCLC who underwent pre-treatment chest CT and EGFR mutation testing were retrospectively enrolled. Tumor volumes of interest (VOIs) were manually segmented on CT images, and radiomic features were extracted using the pyradiomics package. Clinical and radiomic features were selected through a feature selection pipeline, and multiple machine learning algorithms were evaluated for EGFR mutation prediction. Model performance was assessed using the Area Under Curve (AUC).

**Results:**

Predictive performance varied across feature selection strategies and machine learning algorithms. Among all evaluated combinations, the Linear Regression (LR) model built using the Least Absolute Shrinkage and Selection Operator (LASSO)-30 feature set achieved the best performance, with a test-set AUC of 0.745. In this model, CT radiomic features served as the primary predictive component, while selected clinical variables provided complementary information and modestly improved predictive performance. These findings support the value of integrating radiomic and clinical features for non-invasive EGFR mutation prediction in early-stage NSCLC.

**Conclusions:**

A CT radiomics based model demonstrated only moderate performance for the non-invasive prediction of EGFR mutation status in patients with stage IA NSCLC. When clinical variables were incorporated, predictive performance improved, suggesting that clinical features provide complementary information beyond radiomics alone. The combined model highlights the added value of integrating CT-derived radiomics with clinical data for more accurate individualized molecular assessment, particularly when tissue-based genotyping is unavailable or limited.

## Introduction

Lung cancer remains a major global health threat due to its high incidence and mortality rates, and it is the leading cause of cancer-related deaths worldwide (Kratzer et al., 2024). Among all types, non-small cell lung cancer (NSCLC) is the most common, accounting for approximately 80–85% of cases (Islami et al., 2021; Miller et al., 2022). Traditional platinum-based chemotherapy regimens yield a median overall survival (OS) of only 8 to 13 months (Schiller et al., 2002; Ciuleanu et al., 2009), which falls short of current expectations for patient outcomes. With advancements in genetics and molecular biology, increasing evidence suggests that genetic susceptibility to NSCLC is closely associated with a variety of genes and genetic alterations (Liu et al., 2020). Pathogenic mutations in genes such as BRCA2, CHEK2, ATM, NTRK1, EXT2, BRIP1, and PALB2 have been shown to significantly correlate with increased NSCLC susceptibility. These findings offer promising biomarkers and research directions for personalized prevention and treatment strategies.

In NSCLC, major driver mutations such as those in EGFR, KRAS, and ALK activate key signaling pathways (including RAS/RAF/MEK/ERK and PI3K/AKT/mTOR) which play critical roles in tumor cell proliferation, survival, and metastasis. Among these, mutations in the EGFR gene, which encodes a receptor tyrosine kinase, are among the most prevalent driver alterations. EGFR mutations are particularly common in Asian populations, females, and non-smokers (Chan & Hughes, 2015).

The emergence and clinical application of molecular targeted therapies, especially EGFR tyrosine kinase inhibitors (EGFR-TKIs), have significantly reduced mortality rates in lung cancer patients. Meanwhile, the widespread use of low-dose computed tomography (LDCT), along with increased public health awareness and the influence of the pandemic, has led to a significant rise in early-stage lung cancer detection rates.

Currently, for stage I NSCLC patients, clinical guidelines do not provide clear recommendations for adjuvant therapy, particularly in stage IA patients. This is largely due to the relatively low recurrence rate (<30%) in this subgroup and the difficulty in observing statistically significant differences in survival outcomes between those who receive adjuvant therapy and those who do not. Detecting such differences would require large sample sizes. Therefore, routine adjuvant therapy is not currently recommended for stage IA patients. However, this does not imply that adjuvant therapy lacks clinical significance for this patient group.

In the era of conventional chemotherapy, the clinical benefit of adjuvant therapy for stage I NSCLC patients was difficult to demonstrate due to its limited efficacy. However, with the advent of more effective treatment strategies, the potential value of adjuvant therapy in early-stage patients is becoming more observable in clinical studies. For instance, exploratory analyses from the ADAURA investigation (Wu et al., 2020) have shown that adjuvant targeted therapy significantly reduces the risk of recurrence in patients with stage IB NSCLC.

For patients with stage IA there is currently no direct clinical evidence supporting the use of adjuvant targeted therapy. Nonetheless, with the increasing adoption of CT-based lung cancer screening, early detection and surgical resection of pulmonary nodules have become more common, with approximately 90% of these cases being diagnosed as stage IA lung adenocarcinoma. Some studies have suggested that, in EGFR-positive stage IA patients, adjuvant targeted therapy can provide clear benefits. One study reported that, after adjusting for other factors, adjuvant EGFR-targeted therapy improved the 5-year disease-free survival (DFS) rate from 84.5% to 100% (Jiang et al., 2023). Additionally, other studies have demonstrated that EGFR mutations are significantly associated with worse recurrence-free survival (RFS) in stage IA NSCLC (Ito et al., 2020; Kondo et al., 2022; Yang et al., 2024; Huang et al., 2025).

For patients who are elderly or unable to undergo surgery or radiotherapy due to comorbidities, tissue acquisition is typically performed *via* bronchoscopy or percutaneous biopsy. However, both methods carry drawbacks such as invasiveness, longer procedure time, higher cost, dependence on operator skill, and limited sample volume factors that can hinder successful genotyping. In contrast, imaging techniques offer noninvasive, fast, repeatable alternatives. With the rapid development of artificial intelligence (AI), multimodality has emerged as a promising tool that may eventually replace invasive procedures as a primary method for disease characterization.

Previous studies have shown that radiomic features extracted from CT images are associated with EGFR gene expression and can predict EGFR mutation status in NSCLC (Zhang et al., 2022). However, most existing radiomics based studies do not restrict the analysis to specific lung cancer stages or subtypes. In advanced or stage IV NSCLC, the presence of metastases, large primary tumors, or complications such as bronchial obstruction and post-obstructive pneumonia can introduce confounding factors that negatively impact the performance of machine learning models. Studies have also shown the relationship between some clinical features and the inflammation-coagulation axis and lung cancer. For example, some studies have confirmed that high postoperative NLR in patients with solid tumors is associated with worse OS and DFS (Kumarasamy et al., 2019). Similarly, in patients with stage III non-small cell lung cancer treated with radiotherapy and chemotherapy, NLR ≥ 3.57 was found to be independently associated with OS (Tong et al., 2017). However, the relationship between inflammation-related markers and EGFR mutation status has been less extensively investigated.

To minimize such confounding effects, the present study focuses specifically on stage IA non-squamous NSCLC patients. The aim is to evaluate multiple machine learning approaches that integrate CT-based radiomic features and clinical data for predicting EGFR mutation status. This work intends to identify the most effective predictive strategy and provide a foundation for developing individualized treatment plans, as well as for future research into more complex clinical scenarios such as other cancer stages and multiple primary lung cancers.

In recent years, the noninvasive diagnostic paradigm that combines radiomics with clinical variables has made significant progress in predicting EGFR mutations in NSCLC. Early research focused on using relevant biomarkers such as genomic data (Jansen et al., 2018), clinical indicators (Zhang et al., 2019), PET imaging (Deng et al., 2025), and CT scans (Dong et al., 2022). With the advancement of deep learning models, researchers have integrated machine learning to accomplish more complex downstream tasks, including the prediction of EGFR and KRAS mutation status (Chen et al., 2024), as well as survival outcome estimation (Lu et al., 2023).

To further enhance noninvasive patient evaluation, several studies have applied CT-based radiomics and deep learning models for EGFR mutation prediction (Rossi et al., 2021; Nishino et al., 2023; Kim et al., 2024; Le et al., 2025), drug response assessment (Xu et al., 2024), and prognosis estimation (Song et al., 2016). To leverage the strengths of different data types and compensate for the limitations of single-modality inputs, multimodal models have gained increasing attention. These include studies that combine CT-derived features with clinical variables (Ortiz et al., 2022; Kim et al., 2024), further extended by integrating tumor-specific features (Hao et al., 2024), and others that explore the fusion of different imaging modalities such as PET and CT (Xiao et al., 2023).

While large-scale multimodal datasets have shown promising results in EGFR mutation prediction (Zhang et al., 2018), the growing volume and dimensionality of multimodal features often pose challenges in terms of model simplification and interpretability. Additionally, emerging research has revealed that certain cancer developments are closely associated with coagulation and inflammatory markers (Zhou et al., 2023), and these biomarkers can be integrated into machine learning models to provide further insights into disease mechanisms and progression. Because the clinical variables incorporated in previously reported multimodal models have varied substantially, the present study included all routinely available clinical indicators that could be collected from standard examinations. These variables were subsequently subjected to feature selection to systematically determine their individual contributions to EGFR mutation prediction and to clarify which clinical factors provide the greatest complementary value to CT radiomic features.

To advance precision medicine and improve patient care, early screening and risk stratification are especially critical. This study focuses on stage IA NSCLC patients, aiming to develop a predictive model for early identification of individuals with potential EGFR mutations, thereby enabling timely, personalized interventions and proactive risk management.

## Materials and Methods

### Dataset

**Figure 1:**
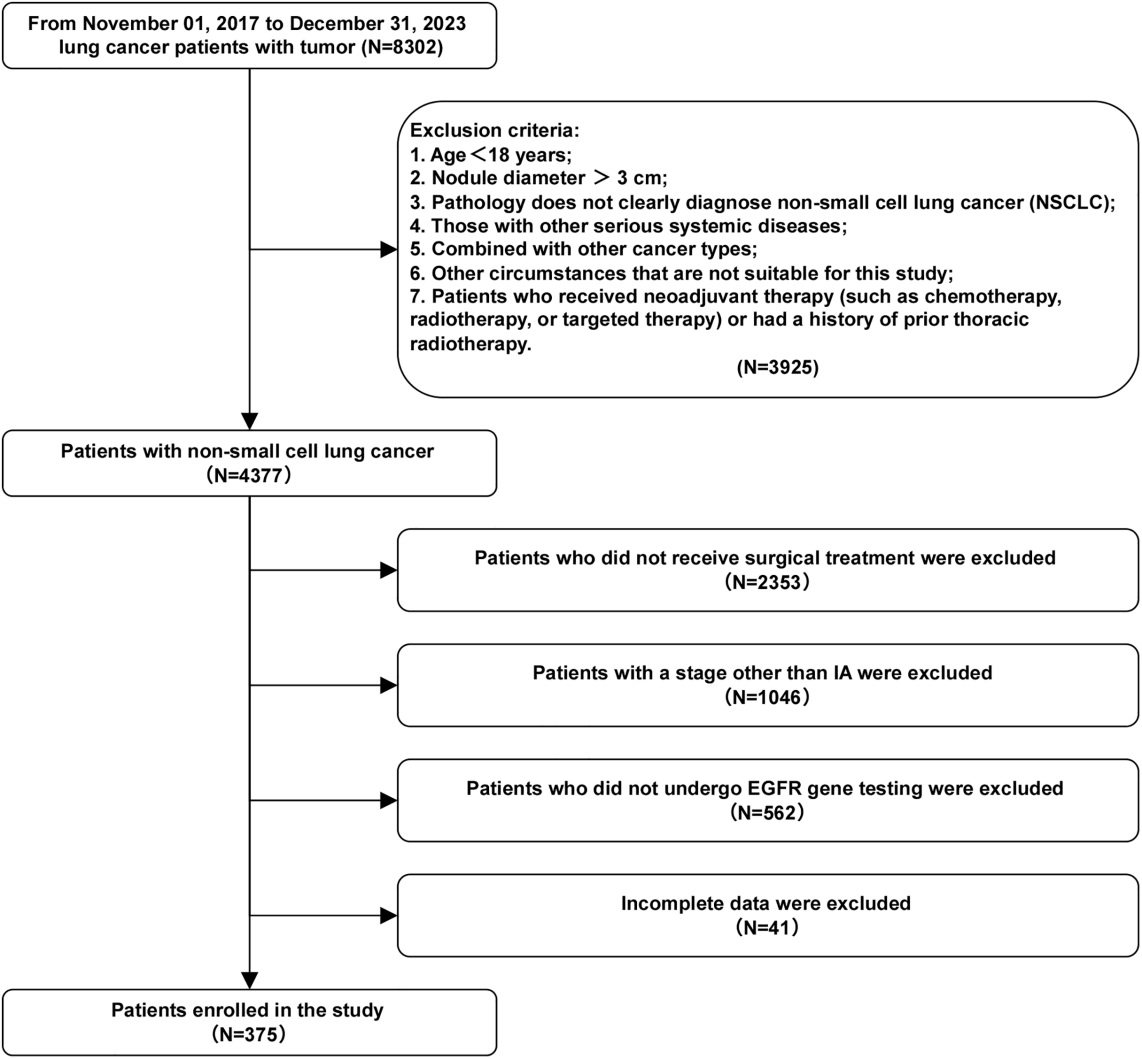
Diagram of the patient selection process.

A retrospective cohort was constructed by collecting records of patients who were diagnosed with lung cancer at Jiangmen Central Hospital between November 1, 2017, and December 31, 2024. A total of 8,302 patient records were initially reviewed. Based on the exclusion criteria (Fig. 1), 3,925 patients with non-adenocarcinoma or unspecified pathological subtypes were excluded. An additional 2,353 patients who did not undergo surgical treatment were removed, followed by the exclusion of 1,046 patients whose postoperative staging was not stage IA. Furthermore, 562 patients without EGFR testing were excluded, and 41 cases with missing CT scans or severely incomplete data were also removed. Ultimately, 375 NSCLC patients who underwent EGFR mutation testing and had pre-treatment CT scans were included in the final analysis.

Clinical data collected included sex, age, height, weight, smoking history, serum biomarkers, tumor markers, inflammatory indicators, and clinical stage. Among the 375 patients, 211 were found to harbor EGFR mutations, while 164 had wild-type EGFR. This study was approved by the Institutional Review Board (IRB) of Jiangmen Central Hospital (Approval No. 2025-232). Given the retrospective nature of the study, the IRB waived the requirement for informed consent from participants. All methods were conducted in accordance with the Declaration of Helsinki.

During data preprocessing, categorical variables were transformed into binary variables using one-hot encoding. For example, sex was encoded as 1 for male and 0 for female, and smoking history was encoded as 1 for smokers and 0 for non-smokers. Numeric variables such as body mass index (BMI), neutrophil-to-lymphocyte ratio (NLR), and platelet-to-lymphocyte ratio (PLR) were derived from other available clinical features. Specifically, NLR was calculated as the absolute neutrophil count divided by the absolute lymphocyte count, while PLR was calculated as the platelet count divided by the absolute lymphocyte count.

Some clinical data had missing values. Features with more than 20% missing data were excluded from the analysis. For the remaining features, all rows containing missing values were temporarily removed to build a linear regression model, which was then used to impute missing values based on the mean-predicted estimates.

### EGFR mutation testing

Tumor tissue specimens were obtained during surgery, and the EGFR mutation status was systematically assessed using high-sensitivity ARMS-PCR (Amplification Refractory Mutation System Polymerase Chain Reaction) technology. The testing procedure involved several steps: First, a pathologist identified and marked tumor cell–rich regions under a microscope to ensure accurate sampling. Second, High-quality genomic DNA was then extracted from the selected areas. Three laboratories independently conducted parallel testing using their respective ARMS-PCR kits: those from Hybribio, MyGene, and the Department of Pathology at Jiangmen Central Hospital. Finally, Amplification and mutation detection were performed on real-time fluorescent PCR instruments to ensure result accuracy, reproducibility, and inter-laboratory consistency.

The detection panel covered exons 18, 19, 20, and 21 of the EGFR gene, allowing simultaneous identification and quantification of both common and rare EGFR mutations. These included, exon 19 deletions (19-Del), exon 21 L858R point mutations, exon 20 T790M resistance mutations, and exon 18 G719X compound mutations *etc*. The process ultimately yielded a comprehensive and reliable EGFR mutation profile report, which served as a molecular pathological basis for subsequent treatment planning and research analysis.

### Clinical variable collection and statistical analysis

Statistical screening of baseline clinical variables and serum tumor markers were collected for all patients, including demographic information (age, sex, height, weight, smoking history), hematological parameters (white blood cell count, neutrophil, lymphocyte, platelet), coagulation indices (prothrombin time (PT), international normalized ratio (INR), fibrinogen (Fib), activated partial thromboplastin time (APTT), D-dimer (Ddi), thrombin time (TT)), biochemical markers (neuron-specific enolase (NSE), carcinoembryonic antigen (CEA), cytokeratin 19 fragment antigen 21-1 (Cyfra21-1)), and derived ratios (NLR, PLR, BMI).

For continuous variables with normal distribution, data are presented as mean ± standard deviation (SD), and between-group comparisons are performed using the Student’s t-test. Variables with non-normal distribution are presented as median and interquartile range (IQR), and comparisons between groups are conducted using the Mann–Whitney U test. Categorical variables are expressed as frequencies and percentages, and comparisons are performed using the chi-square test or Fisher’s exact test, as appropriate.

### CT image acquisition and feature extraction

CT imaging data were acquired using various scanners, including models such as SOMATOM Force, Optima C680 Expert, Ingenuity Core 128, Revolution ACTs, Revolution Ace ES, and Incisive CT Power. The scan range extended from the lung apex to the upper margins of both adrenal glands, ensuring complete coverage of the lung parenchyma. Scanning parameters were standardized across devices: tube voltage was fixed at 120 kVp with automatic tube current modulation enabled *via* CareDose 4D (reference mAs set to 100), collimation was 192 × 0.6 mm, pitch was 0.9, rotation time was 0.5 s, and the image matrix was 512 × 512. Slice thickness was 0.75 mm with an interslice gap of 0.7 mm.

Axial images were reconstructed using both a standard algorithm (Br36) and a lung kernel (Bl57) at 0.75 mm slice thickness. In addition, multiplanar reconstructions (MPR) in axial, coronal, and sagittal planes were performed, with a reconstruction slice thickness and interval of 3 mm and a secondary reconstruction matrix of 512.

Chest CT images in DICOM format were imported into 3D Slicer version 5.6.2 for processing. All cases were reviewed using standardized window settings: lung window (window level: –600 HU; window width: 1,500 HU) and mediastinal window (window level: 40 HU; window width: 400 HU), observed in parallel.

Two pulmonologists each with more than 5 years of experience in thoracic imaging, independently and manually delineated the tumor VOIs on a slice-by-slice basis using a single-blinded protocol (blinded to clinical and pathological data). The Segment Editor module was then used to generate a three-dimensional VOIs for each case.

To assess segmentation consistency, a random subset of 30 lesions was selected to calculate the Dice Similarity Coefficient (DSC) and Hausdorff Distance (HD). If the DSC was below 0.80 or the HD exceeded 5 mm, a third senior pulmonologists with more than 10 years of experience served as an adjudicator. Final consensus VOIs were determined based on majority agreement among the three experts.

Feature Extraction and Preprocessing: Radiomic features were extracted from all VOIs using PyRadiomics 3.1.1 (Python 3.12). The parameters were configured as follows: voxel resampling to 1 × 1 × 1 mm and gray level discretization with 25 bins. In addition to the original images, wavelet transforms (eight sub-bands) and Laplacian of Gaussian (LoG) filters (σ = 1–5 mm, in 1 mm increments) were applied, resulting in six sets of filtered images. The extracted feature classes included: Shape (3D, 14 features), Shape (2D, 10 features), First-order statistics (18 features), Gray Level Co-occurrence Matrix (GLCM, 24 features), Gray Level Size Zone Matrix (GLSZM, 16 features), Gray Level Run Length Matrix (GLRLM, 16 features), Neighboring Gray Tone Difference Matrix (NGTDM, five features), and Gray Level Dependence Matrix (GLDM, eight features). A total of 1,288 features were extracted after all transformations and exported in CSV format for subsequent modeling and analysis.

### Feature selection

**Figure 2:**
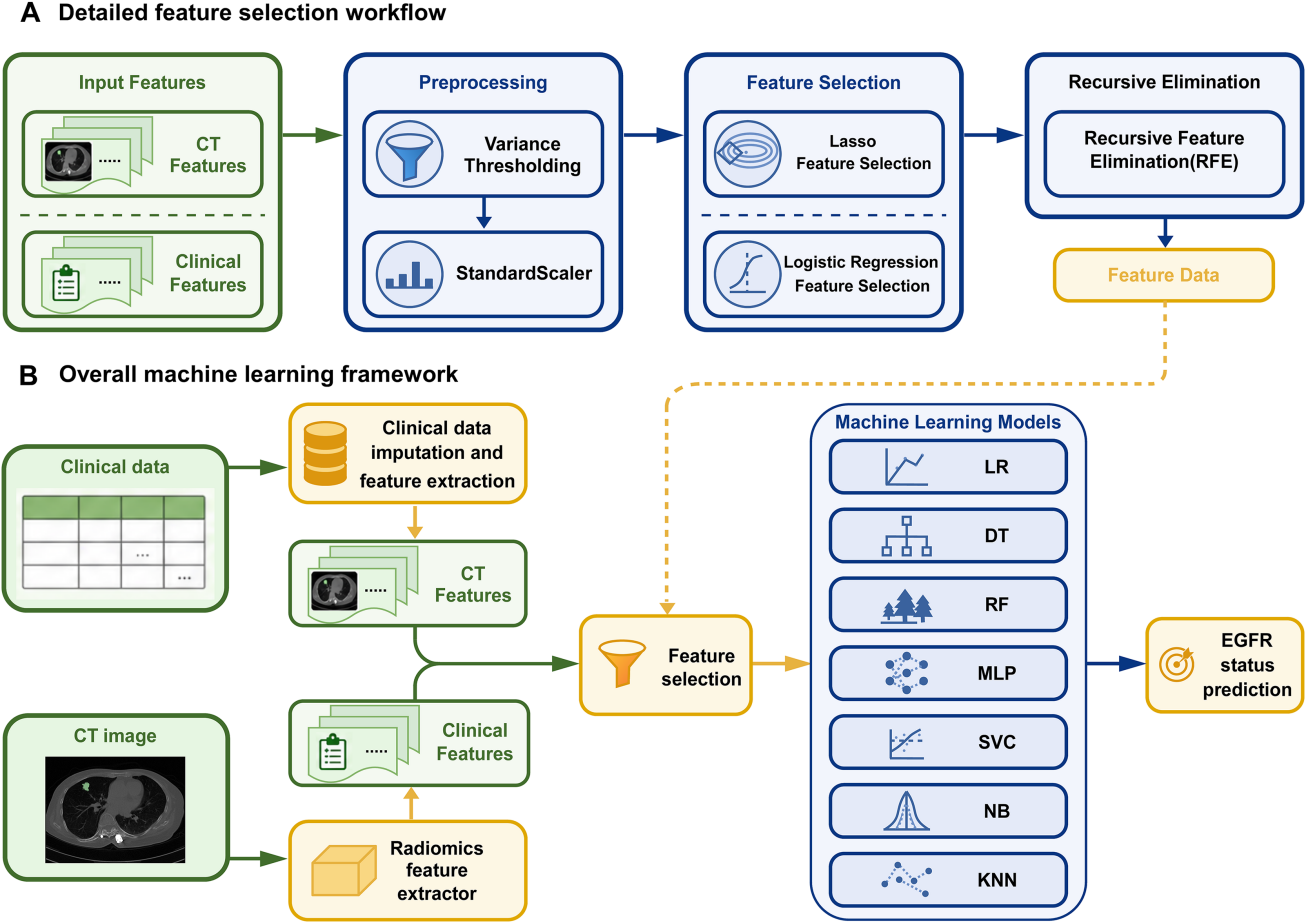
Workflow for EGFR status prediction with clinical and CT radiomics features. (A) Detalled workfiow of the feature selection procedure, including variance thresholding, standardization, LASSO, logistic regresion-based selection, and recursive feature elimination (RFE). (B) Overal framework for integrating clinical and CT radiomics features for EGFR status prediction. The details of feature selection are in A.

A three-level feature selection strategy, as illustrated in Fig. 2A, was employed in this study. Features with low variance were removed using a Variance Threshold (Threshold = 0.01) to eliminate constant features, while avoiding correlation-based filtering to preserve potential nonlinear relationships. The remaining features were then standardized using z-score normalization. Two parallel methods, Least Absolute Shrinkage and Selection Operator (LASSO) (λ = 0.01) and logistic regression with L1 regularization were applied to obtain feature subsets, retaining only those features with non-zero absolute coefficients in each method.

To evaluate the influence of different feature selection strategies on downstream model performance, multiple feature subsets were constructed and compared. These included: (1) directly selected features without Recursive Feature Elimination (RFE); (2) the top 20, 25, and 30 features selected after RFE; and (3) feature subsets derived exclusively from CT radiomic features to further investigate the contribution of clinical variables to model performance. In addition, LASSO with cross-validation (LASSO-CV) was independently applied as an alternative feature selection approach to validate the robustness and effectiveness of the selected feature sets.

Finally, the resulting feature subsets were incorporated into subsequent predictive modeling and validation procedures to systematically assess the sensitivity and performance of the models under different feature selection strategies.

### Model construction and training

After feature selection, multiple feature subsets were used to develop and evaluate predictive models using a series of machine learning algorithms, as illustrated in Fig. 2B. To comprehensively assess the influence of different feature selection strategies on model performance, each feature subset was independently applied to multiple machine learning classifiers. The evaluated models included Linear Regression (LR), Decision Tree (DT), Random Forest (RF), Multilayer Perceptron Multilayer Perceptron (MLP), Support Vector Classification (SVC), Naive Bayes (NB), and k-Nearest Neighbors (KNN).

Model validation were conducted using 10-fold stratified cross-validation (StratifiedKFold) to maintain class distribution consistency across folds. In each fold, 90% of the data were used for training and the remaining 10% for testing. Performance metrics were calculated for each fold, and the mean values together with 95% confidence intervals (95% CI) were subsequently recorded.

Default hyperparameters were used where possible to ensure consistency. For models that failed to converge or exhibited signs of overfitting, adjustments were implemented by increasing the maximum number of iterations and applying Cost-Complexity Pruning with a pruning parameter of 0.01. To ensure reproducibility, all models were configured with random_state = 0.

Each model presents distinct strengths: LR offers high interpretability under the linearity assumption; DT are intuitive but prone to overfitting; RF reduce variance through bagging and are robust to high-dimensional data; MLP capture complex nonlinear relationships *via* neural network architectures but require extensive training and parameter tuning; SVC perform well in high-dimensional spaces using kernel functions, albeit with increased computational cost for large datasets; NB is efficient under the assumption of feature independence and is stable in small-sample scenarios; and KNN makes predictions based on local instance similarity without explicit training, although it is sensitive to dimensionality and computational burden.

### Evaluation metrics

The performance of the classification models was evaluated using a set of objective metrics, including accuracy, precision, recall, specificity, sensitivity, F1-score, and AUC. Several of these metrics were calculated based on the model predictions of true positives (TP), true negatives (TN), false positives (FP), and false negatives (FN). Additionally, the AUC of the ROC curve was used to assess the binary classification performance of deep learning algorithms. To plot the ROC curve, the true positive rate (TPR) and false positive rate (FPR) were recorded at various probability thresholds. All statistical evaluations were two-tailed, and a *P*-value < 0.05 was considered statistically significant throughout the analysis.

All computational analyses were conducted on Windows 11 using Python 3.12, with the following libraries and frameworks: OpenCV-Python (version 4.10.0.84), Pandas (version 2.2.2), Scikit-learn (version 1.6.1), NumPy (version 1.26.4), Matplotlib (version 3.8.4), and PyTorch (version 2.5.0). The modeling and training were performed on a server equipped with an NVIDIA GeForce RTX 4090D GPU and an Intel i9-14900KF CPU.

## Results

### Clinical features

As shown in Table 1, among the 375 patients included in the study, 164 were EGFR wild-type and 211 were EGFR mutant-type. There were no statistically significant differences between the wild-type and mutant groups in terms of demographic variables (age, height, weight, BMI), routine blood cell counts (white blood cells, neutrophils, lymphocytes, platelets), coagulation parameters (INR, fibrinogen, APTT, D-dimer), tumor markers (CEA, Cyfra21-1), and smoking history (*P* > 0.05). Only three variables reached statistical significance: neuron specific enolase (NSE), prothrombin time (PT), and thrombin time (TT). The median NSE level in the mutant group was 11.54 ng/mL, lower than that in the wild-type group (12.09 ng/mL; Z = –2.455, *P* = 0.014). The mean PT in the wild-type group was slightly higher (11.1 s) compared to the mutant group (11.0 s; t = –2.055, *P* = 0.04), and the mean TT in the mutant group was slightly longer (17.16 s) than in the wild-type group (16.88 s; t = –2.512, *P* = 0.012). These findings suggest minor differences in neuroendocrine activity and terminal coagulation time between the two groups. However, the magnitude of these differences was small and of limited clinical relevance. Overall, the baseline clinical features were well-balanced between the two groups, indicating that the subsequent model performance was primarily driven by radiomic features, rather than conventional clinical variables.

**Table 1:** Clinical features and serum tumormarkers of all patients.

Variables	EGFR-wild (*n* = 164)	EGFR-mutant (*n* = 211)	*Z*	*P*-value
Age	61 (52, 69)	63 (55, 70)	−1.488	0.137
Height	160 (155, 166)	158 (155, 165)	−1.508	0.132
Weight	58 (51.13, 67)	57 (51, 64)	−1.009	0.313
white_blood_cell	6.36 (5.33, 7.29)	6.32 (5.19, 7.56)	−0.193	0.847
Neutrophil	3.75 (3.05, 4.59)	3.8 (2.88, 5)	−0.361	0.718
Lymphocyte	1.84 (1.48, 2.27)	1.76 (1.39, 2.16)	−1.705	0.088
Platelet	237 (206.25, 274)	234 (203, 271)	−0.175	0.861
CEA	2.33 (1.49, 3.96)	2.12 (1.41, 3.55)	−1.278	0.201
Cyfra21-1	2.32 (1.63, 3.51)	2.17 (1.49, 3.31)	−1.068	0.286
NSE	12.09 (10.57, 13.75)	11.54 (10.06, 12.91)	−2.455	**0.014**
PT	11.1 (10.8, 11.58)	11 (10.5, 11.5)	−2.055	**0.04**
INR	0.93 (0.87, 0.97)	0.93 (0.88, 0.96)	−0.593	0.553
Fib	2.81 (2.36, 3.09)	2.75 (2.38, 3.12)	−0.387	0.699
APTT	26.6 (24.13, 28.48)	26.1 (24.1, 28.2)	−0.667	0.505
Ddi	0.28 (0.16, 0.49)	0.26 (0.17, 0.55)	−0.175	0.861
NLR	2.04 (1.52, 2.56)	2.07 (1.57, 2.93)	−1.18	0.238
PLR	125.98 (98.67, 154.29)	133.6 (106.49, 170.49)	−1.88	0.06
TT	16.88 ± 1.16	17.16 ± 1.08	−2.512	**0.012**
BMI	22.97 ± 3.61	22.84 ± 3.36	0.335	0.738
Sex			0.001	0.975
Female	100 (61.0)	129 (61.1)		
Male	64 (39.0)	82 (38.9)		
Smoking			0.861	0.353
No	124 (75.6)	168 (79.6)		
Yes	40 (24.4)	43 (20.4)		

**Note:**

Clinical features and serum tumormarkers were compared between patients with EGFR-wild tumors (*n* = 164) and those with EGFR-mutant tumors (*n* = 211). Continuous variables are presented as mean ± standard deviation (SD) for normally distributed data or median (interquartile range, IQR) for non-normally distributed data. Categorical variables are presented as number (percentage). Group comparisons were performed using Student’s t-test, Mann–Whitney U test, chi-square test, or Fisher’s exact test, as appropriate. The Z column represents the test statistic for nonparametric comparisons, and statistically significant *P* values (*P* < 0.05) are shown in bold. Abbreviations: CEA, carcinoembryonic antigen; Cyfra21-1, Cytokeratin 19 Fragment Antigen 21-1; NSE, neuron-specific enolase; PT, prothrombin time; INR, international normalized ratio; Fib, fibrinogen; APTT, activated partial thromboplastin time; Ddi, D-dimer; NLR, neutrophil-to-lymphocyte ratio; PLR, platelet-to-lymphocyte ratio; TT, thrombin time; BMI, body mass index.

### Molecular characteristics

**Figure 3:**
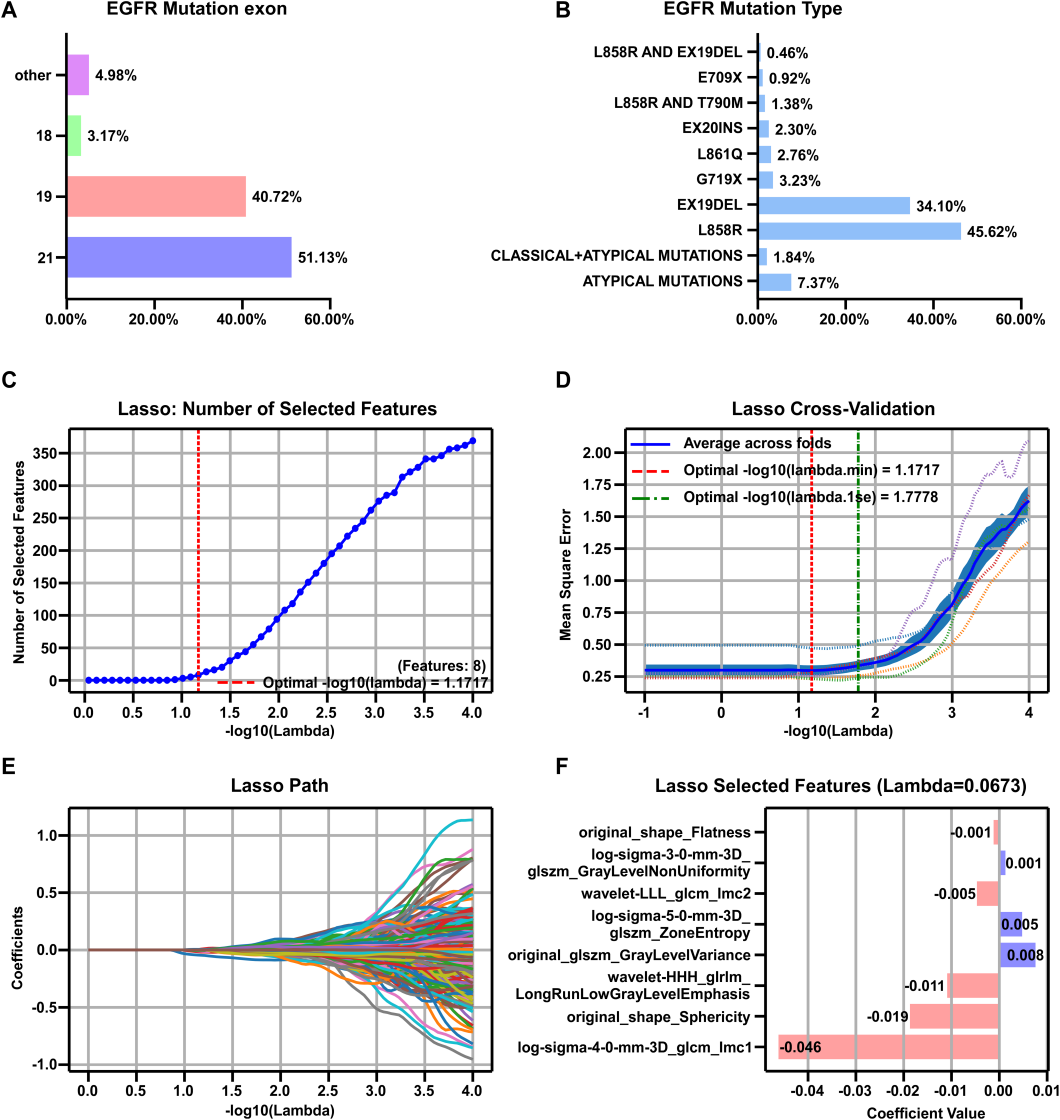
Analysis of EGFR mutations and feature selection *via* LASSO regression. (A) EGFR Mutation Exon Distribution, This bar chart illustrates the distribution of EGFR mutations across different exons. (B) EGFR Mutation Type Proportions, This bar chart shows the proportion of different types of EGFR mutations. (C) LASSO: Number of Selected Features, This plot depicts the number of features selected by LASSO regression as the regularization parameter −log_10_(λ) varies. (D) LASSO Cross-Validation, This plot shows the mean square error (MSE) across different folds of cross-validation for varying −log_10_(λ) values. (E) LASSO Path, This plot illustrates the path of coefficients as −log_10_(λ) increases. (F) LASSO Selected Features, This heatmap displays the coefficients of the features selected by LASSO at λ = 0.0673.

Figures 3A and 3B illustrate the distribution of EGFR gene mutations. As shown in Fig. 3A, the mutations are distributed across different EGFR exons, with exon 21 being the most frequently mutated (51.13%), followed by exon 19 (40.72%). Mutations in exon 18 accounted for 3.17%, while mutations in other exons represented only 4.98%.

Figure 3B provides a more detailed breakdown of specific EGFR mutation types. The most common mutation was L858R, accounting for 45.62%, followed by exon 19 deletions (EX19DEL) at 34.10%. Other mutation types including G719X, L861Q, EX20 insertions, co-occurrence of L858R and T790M, co-occurrence of L858R and EX19DEL, and E709X, each accounted for less than 3%. Additionally, classical mutations combined with atypical mutations comprised 1.84%, while atypical mutations alone accounted for 7.37%.

Overall, EGFR mutations were primarily concentrated in exons 19 and 21, with L858R and EX19DEL being the most prevalent types. These findings are of critical importance for guiding EGFR-targeted therapies.

### Association between clinical and CT features

To ensure the accuracy of feature coefficient selection, LassoCV was first applied to determine the appropriate regularization strength (α) for feature selection. As shown in Fig. 3C, the number of selected features gradually decreased as the regularization parameter increased. When −log_10_(λ) = 1.1717, the number of retained features dropped to eight, representing the optimal feature subset.

Figure 3D displays the mean squared error (MSE) across different regularization levels. The lowest MSE was observed when −log_10_(λ) was in the range of 1.1717 to 1.1778, indicating that λ values within this range yielded optimal model performance. This corresponds to an α value on the order of 0.01, which was therefore chosen for LASSO-based feature selection.

Figure 3E presents the LASSO path, illustrating how the coefficients of various features changed with increasing −log_10_(λ). As regularization strength increased, more feature coefficients shrank to zero, indicating progressive feature elimination.

Figure 3F shows the eight features selected at the optimal λ = 0.0673, along with their corresponding coefficient values. Among these, five features had negative coefficients and three had positive coefficients. The selected features included three derived from original CT images, two from wavelet-transformed images, and three from Laplacian of Gaussian (LoG)-filtered images.

**Figure 4:**
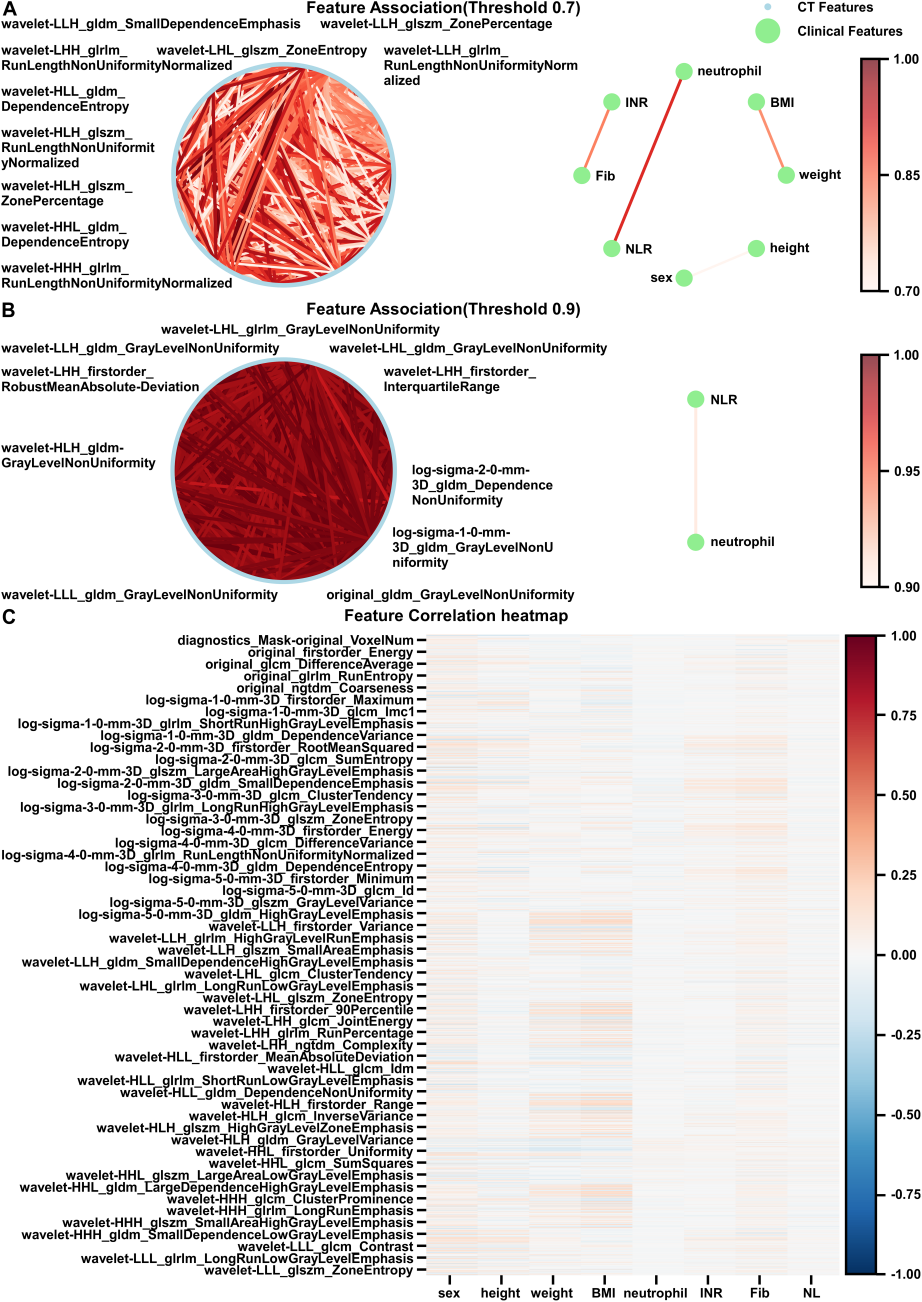
Feature association and clinical relevance. (A, B) Self-organizing association networks constructed using absolute Pearson correlation thresholds of 0.70 and 0.90. Radiomic features are displayed on the left and clinical variables on the right. Only feature nodes and edges with correlation coefficients exceeding the specified threshold are shown, features with correlations below the threshold are omitted. The names of the top 10 radiomic features with the strongest correlations are labeled for clarity. Edge color intensity reflects correlation strength, with darker red indicating stronger positive correlations. (C) Heatmap of Pearson correlation coefficients between selected CT radiomic features (rows) and clinical variables (columns). Red indicates positive correlations, blue indicates negative correlations, and color intensity reflects the strength and direction of the correlations.

Exploring the relationship between CT and clinical features, to gain a deeper understanding of the correlation between CT-based radiomic features and clinical variables, Fig. 4 provides an in-depth analysis of both feature interrelationships and clinical associations. As shown in Fig. 4A, a feature correlation network was constructed using a threshold of 0.7 to identify significant connections among features. The network highlights the top 10 CT features with the highest number of connections, while only clinically relevant features that exhibit non-negligible correlations are included. The analysis revealed that there were no strong direct correlations between CT features and clinical variables. Among the CT features, wavelet-transformed features appeared most frequently in the network, although connections were weak (indicated by lighter edge colors), suggesting limited high-strength associations.

In Fig. 4B, a more stringent correlation threshold of 0.9 was applied to identify the most robust relationships. At this level, only a few clinical features, most notably neutrophil count and NLR (neutrophil-to-lymphocyte ratio), remained in the network. This is expected, as NLR is directly calculated from neutrophils, leading to a naturally high correlation. On the radiomics side, low-order Laplacian of Gaussian (LoG) features began to emerge, particularly those related to Gray Level Non-Uniformity, indicating their potential role in capturing robust structural patterns in the images.

Figure 4C presents a heatmap of the correlation coefficients between CT and clinical features, focusing only on pairs with relatively higher correlations. Notably, body weight and BMI showed moderate to strong positive or negative correlations with specific CT features, followed by sex, and to a lesser extent, fibrinogen (Fib). These findings suggest that while most CT and clinical features are largely independent, certain features especially Gray Level Non-Uniformity, wavelet, and LoG-based features, may carry latent biological relevance. This analysis provides important insights into the underlying relationships between imaging and clinical data, and supports the integration of multimodal features in future predictive modeling efforts.

### Differences in feature selection results

**Figure 5:**
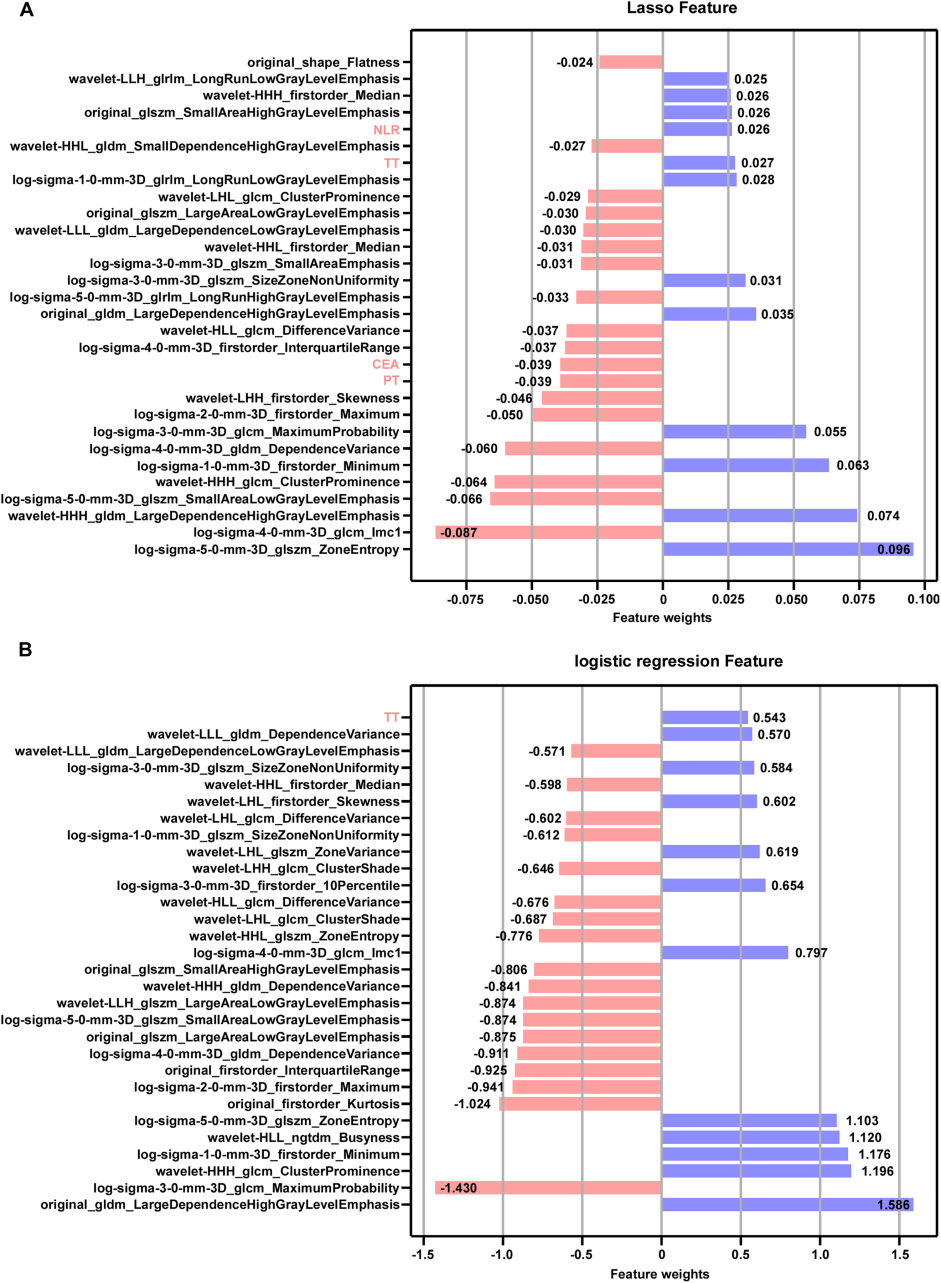
Feature importance in lasso and logistic regression models. (A) LASSO Regression and (B) Logistic Regression Feature Weights, Bar plot illustrating the non-zero coefficients of features selected by the regression model. Blue bars represent features with positive coefficients, indicating a positive association with EGFR mutation, whereas red bars represent features with negative coefficients, indicating a negative association. The x-axis denotes the standardized coefficient weights, and the y-axis lists the selected features. Clinical variables are highlighted in red text.

To gain a more comprehensive understanding of which features consistently demonstrate high predictive value across different models is crucial for constructing an accurate and interpretable predictive model. The results presented in Fig. 5 reveal the top 30 most important features and their corresponding weights in both the LASSO regression and logistic regression models, highlighting the contribution of different features to the prediction outcomes.

The study found that clinical data such as NLR, CEA, prothrombin time (PT), and thrombin time (TT) contributed significantly to the models. However, only TT appeared in the feature selection process of the logistic regression model, suggesting that these clinical indicators may play a key role in disease diagnosis or prognosis evaluation.

For CT image features, the most important features selected by the LASSO regression model were log-sigma-5-0-mm-3D_glszm_ZoneEntropy and log-sigma-4-0-mm-3D_glcm_lmc1. The selected CT features were relatively evenly distributed across different types, including four original image features, 10 wavelet-transformed features, 12 LoG related features, and four clinical features.

In contrast, the most important features selected by the logistic regression model were original_gldm_LargeDependenceHighGrayLevelEmphasis and log-sigma-3-0-mm-3D_glcm_MaximumProbability. The CT features selected in logistic regression showed a proportional distribution across feature types, comprising five original image features, 14 wavelet-transformed features, 10 LoG-related features, and only one clinical feature. These features may reflect certain specific radiological features of the tumor, which are significant for disease identification and classification.

By comparing the feature coefficients of the two models, we observed that both had 18 features with negative weights and 12 with positive weights. The magnitude of feature weights in the Lasso regression model was generally smaller than those in the logistic regression model. Each feature selection method has its own features. For example, Lasso tends to incorporate features from different dimensions more evenly, while logistic regression tends to focus on task-relevant features.

### Model performance comparison

**Figure 6:**
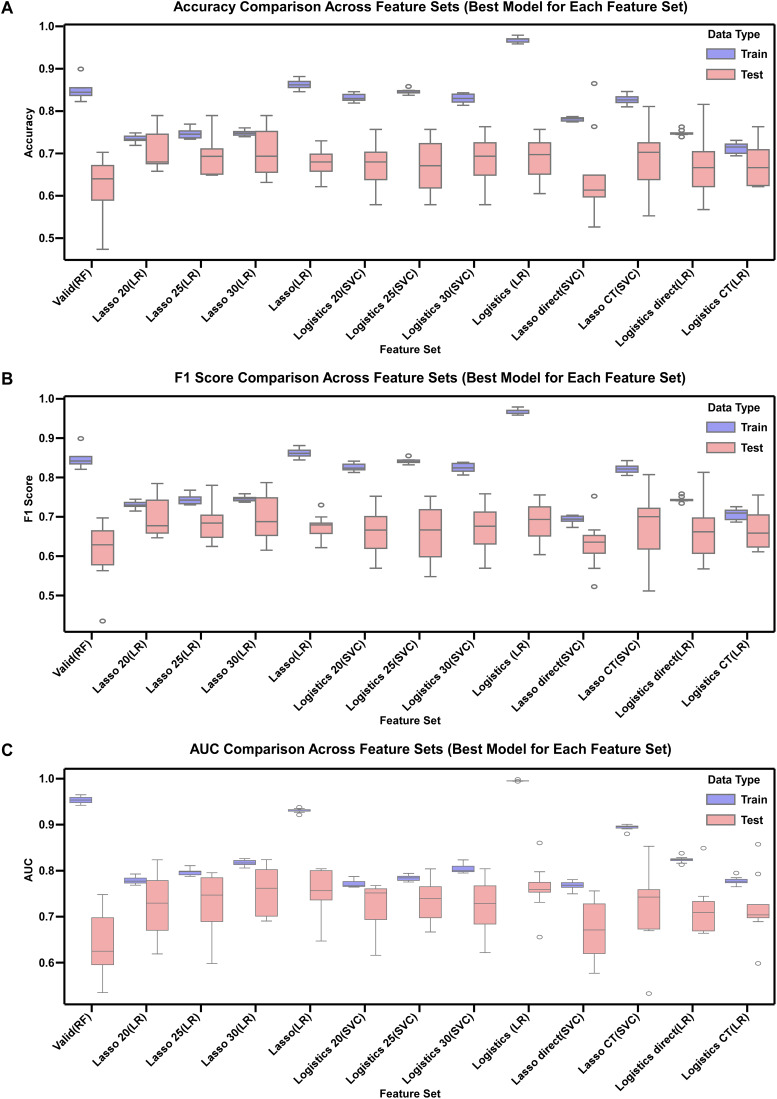
Model performance comparison across feature sets (best model per set). (A) Accuracy Comparison. (B) F1-score Comparison. (C) AUC Comparison. Boxplots showing the distribution of model performance obtained by 10-fold stratified cross-validation for the best-performing classifier within each feature set. Blue boxes represent training performance and red boxes represent test performance. Each feature set corresponds to a distinct feature selection strategy, including features selected directly by LASSO, features further reduced by recursive feature elimination (top 20, 25, or 30 features), CT-only feature subsets, and feature subsets selected using LASSO-CV for validation (Valid). The central line indicates the median, boxes represent the interquartile range and circles indicate outliers.

This study included a total of 375 participants and employed 10-fold cross-validation to evaluate model performance. Key results including accuracy, F1-score, and AUC are shown in Fig. 6 and Table 2. In the figure, the x-axis represents different feature combinations, with the best-performing model for each combination indicated in parentheses. The groups “Lasso 20/25/30” refer to feature subsets obtained by applying Lasso for initial selection, followed by Recursive Feature Elimination (RFE) to retain the top 20, 25, or 30 features, respectively. The “Lasso” group represents features selected directly by Lasso without limiting the number, with RFE applied thereafter; the same logic applies to the “Logistics” group using logistic regression for initial selection.

**Table 2:** Predictive performance of various models in training and validation.

	Training group						Validation group					
	ACC	PRE	SPE	SEN	F1	AUC	ACC	PRE	SPE	SEN	F1	AUC
Valid (RF)	0.847	0.851	0.753	0.921	0.845	0.953	0.624	0.623	0.483	0.734	0.612	0.630
Lasso 20 (LR)	0.734	0.734	0.609	0.832	0.730	0.778	0.704	0.709	0.574	0.805	0.696	0.721
Lasso 25 (LR)	0.746	0.746	0.640	0.828	0.743	0.796	0.693	0.702	0.562	0.796	0.684	0.733
**Lasso 30 (LR)**	**0.748**	**0.747**	**0.651**	**0.824**	**0.745**	**0.817**	**0.703**	**0.712**	**0.587**	**0.795**	**0.695**	**0.745**
Lasso (LR)	0.863	0.863	0.805	0.907	0.862	0.930	0.674	0.683	0.634	0.706	0.672	0.746
Logistics 20 (SVC)	0.831	0.847	0.670	0.956	0.826	0.889	0.677	0.685	0.495	0.820	0.663	0.709
Logistics 25 (SVC)	0.845	0.859	0.696	0.961	0.841	0.908	0.669	0.669	0.495	0.805	0.656	0.707
Logistics 30 (SVC)	0.829	0.845	0.670	0.953	0.824	0.891	0.682	0.687	0.513	0.814	0.672	0.686
Logistics (LR)	0.968	0.968	0.955	0.978	0.968	0.995	0.690	0.694	0.628	0.739	0.688	0.760
Lasso direct (SVC)	0.780	0.788	0.630	0.897	0.775	0.876	0.645	0.643	0.445	0.801	0.629	0.645
Lasso CT (SVC)	0.826	0.841	0.669	0.949	0.821	0.894	0.682	0.683	0.496	0.829	0.667	0.720
Logistics direct (LR)	0.748	0.749	0.620	0.847	0.743	0.823	0.666	0.667	0.542	0.763	0.660	0.714
Logistics CT (LR)	0.712	0.713	0.562	0.829	0.706	0.777	0.676	0.682	0.537	0.786	0.670	0.719

**Note:**

Model performance was evaluated using 10-fold stratified cross-validation. For each feature set, the best-performing machine learning algorithm was selected and its mean performance metrics are reported for the training and validation cohorts. Feature sets included variables selected directly by LASSO, features further reduced by recursive feature elimination (top 20, 25, or 30 features), CT-only feature subsets, and an independent validation feature set selected using LASSO-CV (Valid). Values represent the average performance across the 10 folds. The bold text represents the model and feature set combination that performs best with a limited number of features, and will be used for subsequent analysis and discussion. Abbreviations: ACC, accuracy; PRE, precision; SPE, specificity; SEN, sensitivity; F1, F1-score; AUC, area under the receiver operating characteristic curve; LR, Linear Regression; SVC, support vector classification; RF, random forest; CT, computed tomography.

The labels “Lasso direct” and “Logistics direct” indicate that CT features were selected first, and then clinical variables were directly added to the model input without further recursive filtering. Among the three feature sets containing only CT features as Valid (RF), Lasso CT (SVC), and Logistics CT (LR), it was observed that the Valid group consistently showed lower accuracy on the test set, suggesting that feature selection is highly dependent on the dataset, and that EGFR mutation prediction is a data-driven task. These results highlight the importance of dataset-specific feature selection strategies in achieving optimal model performance.

In the training set, all feature combinations demonstrated excellent performance, with accuracy (ACC), F1-score, and AUC generally exceeding 0.80, indicating a strong fitting ability of the models on internal data. However, performance dropped significantly on the testing set, revealing a clear risk of overfitting. Specifically, LR achieved the highest AUC in the training set 0.968, maintained the highest AUC in the validation set 0.760 among all models. However, it showed the largest performance gap between training and testing sets and involved as many as 174 features, making it less practical for research or model deployment.

In contrast, LASSO (LR) maintained a high AUC in the training set 0.863 and achieved a relatively good performance in the validation set 0.746, making it the best-performing model among all LASSO-based methods. This suggests that while the model is highly dependent on the training data, its ability to generalize is somewhat limited. Nevertheless, the combination of Lasso feature selection and logistic regression appears to help mitigate overfitting to some extent.

Notably, logistic regression consistently emerged as the best-performing model across different feature selection strategies, followed by the SVC, RF only performed best in the validation set. This suggests that the choice of feature selection strategy can influence the optimal downstream model.

Overall, both Lasso (LR) and Lasso 30 (LR) achieved the best performance in the validation set. However, Lasso 30 (LR), which uses only 30 key features, offers a much simpler model compared to the 96-feature Lasso (LR) model, with only minimal differences in performance. Therefore, subsequent analyses will be based on the Lasso 30 (LR) model.

### Relationship between selected features and EGFR mutation

**Figure 7:**
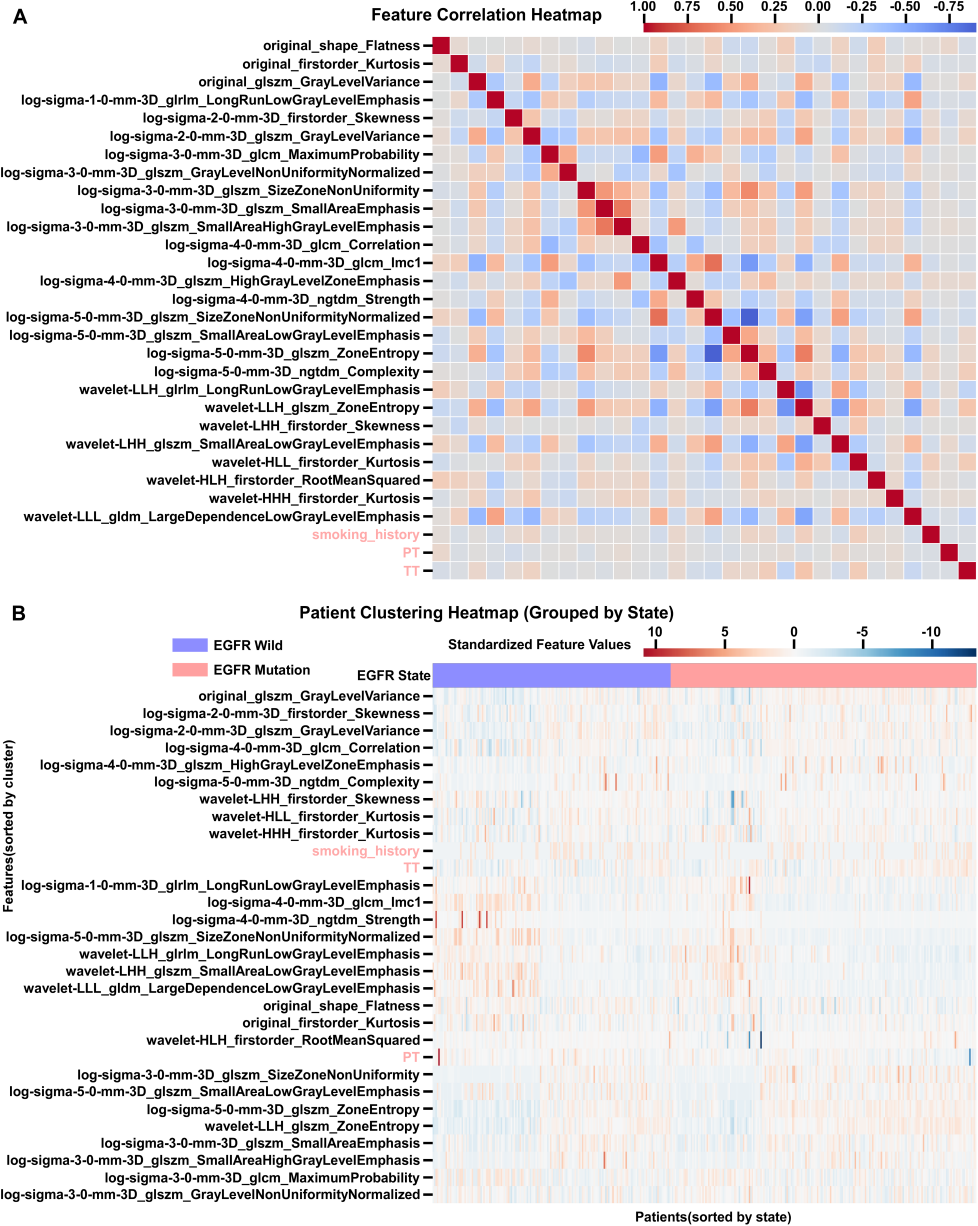
Heatmaps of feature correlation and patient clustering grouped by EGFR status. (A) Correlation heatmap of the features included in the best-performing feature set identified across all feature selection strategies and machine learning models. The feature set contains both CT radiomic and clinical variables, with clinical features highlighted in red text. Color indicates the Pearson correlation coefficient, ranging from −1 (blue) to 1 (red). (B) Heatmap of standardized feature values across all patients. Columns represent patients ordered by EGFR status, and rows represent the selected features. The top annotation bar indicates EGFR-wild (blue) and EGFR-mutant (pink) groups. Clinical variables are highlighted in red text. Red indicates higher standardized values and blue indicates lower values.

After determining the optimal research model, we proceeded to analyze the relationship between the selected features and EGFR mutation status. The Pearson correlation heatmap shown in Fig. 7A illustrates the interactions among the selected features. The color intensity in the heatmap indicates the strength of correlation between features, red represents positive correlation, while blue indicates negative correlation.

The analysis revealed that most features exhibited weak correlations with each other, particularly between radiomic features and clinical features. However, within the radiomic features themselves, some strong positive and negative correlations were observed. Notably, smoking history showed a positive correlation with several radiomic features, which may be related to the impact of smoking on lung tissue structure and function. Additionally, coagulation function indicators were found to be associated with certain radiomic features, suggesting that coagulation may influence the physical density and vascular permeability of tissues surrounding the lesion.

Figure 7B presents an unsupervised clustering heatmap that classifies patients into two groups based on EGFR mutation status: EGFR wild-type (light blue) and EGFR mutant-type (light red). From top to bottom, the heatmap highlights the key differentiating features between the two groups. The most significant radiomic feature identified was original_glszm_GrayLevelVariance, which reflects the variation in gray levels within connected regions on the original CT images, indicating greater intraregional heterogeneity in EGFR mutant cases. Among the clinical features, smoking history emerged as the most significant, further supporting its potential role in the development of EGFR mutations. These findings suggest that EGFR mutation status cannot be accurately determined by any single indicator alone. Instead, a combination of multiple features and machine learning methods is necessary to perform deeper, multi-dimensional data mining for reliable prediction.

### Model performance evaluation

**Figure 8:**
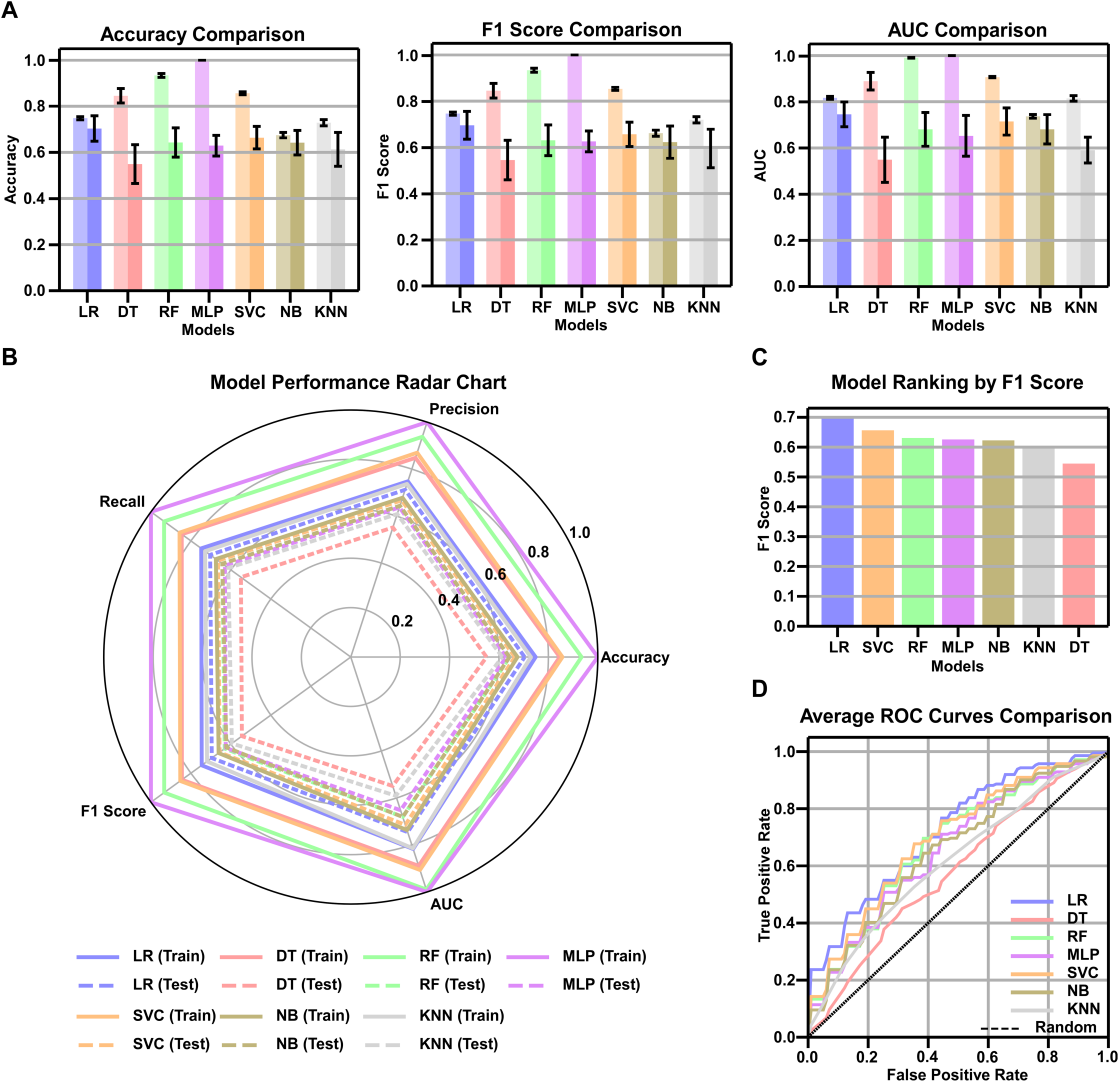
Comprehensive model performance evaluation. (A) Comparison of training and test performance for seven machine learning models, including Linear Regression (LR), Decision Tree (DT), Random Forest (RF), Multilayer Perceptron Multilayer Perceptron (MLP), Support Vector Classification (SVC), Naive Bayes (NB), and k-Nearest Neighbors (KNN). Performance metrics include accuracy, F1-score, and area under the receiver operating characteristic curve (AUC). Bars represent the mean values obtained from 10-fold stratified cross-validation, and error bars indicate the variance across folds. (B) Radar chart summarizing the performance of each model on the training and test sets across five metrics: accuracy, precision, recall, F1-score, and AUC. (C) Ranking of the seven models according to their mean test-set F1-scores. (D) Average receiver operating characteristic (ROC) curves of the seven models on the test sets, with the diagonal dashed line indicating random classification performance.

Subsequently, we applied various machine learning models to the Lasso30 feature subset. The results, as shown in Fig. 8, provide a comprehensive evaluation of the performance of different models using this feature set.

Figure 8A presents a bar chart comparing the performance of seven models LR, DT, RF, MLP, SVC, NB, and KNN. In terms of accuracy, F1-score, and AUC on both the training and testing sets (light-colored bars for training, dark-colored for testing). The MLP model achieved the best performance on the training set but showed clear signs of overfitting. In contrast, LR performed best on the testing set, achieving an AUC of 0.745, indicating strong discriminative ability between classes. Among all models, DT exhibited the largest performance variance, while LR had the smallest variance, suggesting that LR is better suited for this downstream task. It consistently achieved accuracy above 0.7 without signs of overfitting.

Figure 8B presents a radar chart illustrating that both LR and NB performed most consistently across five key metrics: precision, recall, accuracy, F1-score, and AUC. Among them, RF also showed relatively strong performance. Figure 8C ranks the models based on their F1-scores, further highlighting the performance gap between training and testing sets. This suggests that model generalizability still has room for improvement, while LR continues to demonstrate superior and stable performance. Figure 8D compares the ROC curves of all models, showing that LR had the best class discrimination ability, followed closely by SVC. In summary, these results underscore the importance of balancing different performance metrics when selecting a model. Logistic regression demonstrated strong predictive performance and generalizability in the binary classification task, making it a favorable choice in this context.

## Discussion

NSCLC is characterized by complex pathological subtypes and significant heterogeneity, with considerable variation in biological behavior and patient prognosis. The development of molecular targeted therapies has substantially advanced the treatment of NSCLC. Among the molecular targets, the EGFR has attracted the most clinical attention. Approximately 50% of Asian patients with lung adenocarcinoma and 10–15% of Caucasian patients harbor EGFR gene mutations (Chan & Hughes, 2015), with the most common alterations being exon 19 deletions and the exon 21 L858R point mutation (Reguart & Remon, 2015). The introduction of EGFR tyrosine kinase inhibitors (EGFR-TKIs) has brought significant clinical benefits to patients with sensitizing EGFR mutations. Currently, three generations of EGFR-TKIs are available for clinical use. First-generation EGFR-TKIs, such as erlotinib and gefitinib, have demonstrated notable efficacy in patients with classical EGFR mutations (Del19 or L858R), prolonging median progression-free survival (PFS) to 9.8–11 months, significantly longer than that achieved with standard chemotherapy (Wen et al., 2018). Second-generation TKIs, including afatinib and dacomitinib, have shown improved efficacy with median PFS ranging from 11.0 to 14.7 months, although they are associated with a higher incidence of adverse events (Sharma & Graziano, 2018). Third-generation TKIs, such as osimertinib and furmonertinib, have demonstrated superior effectiveness and better safety profiles in first-line treatment of advanced NSCLC with EGFR mutations, with a median PFS of 17.8–20.8 months and grade ≥3 treatment-related adverse events occurring in 11–25% of cases (Soria et al., 2018).

While radical surgery remains the first-line treatment for early-stage lung cancer, the optimal timing for adjuvant therapy remains controversial. Recently, targeted adjuvant therapies have been increasingly introduced in earlier stages, with growing evidence supporting their benefit in patients with EGFR mutated early-stage NSCLC after surgical resection. For patients who are ineligible for surgery due to various reasons, such as limited tissue samples or the risk of invasive diagnostic procedures, radiomics has emerged as a promising alternative. As a non-invasive, convenient, cost-effective, and reproducible approach, radiomics is playing an increasingly important role in clinical practice, offering a new avenue for precision diagnosis and individualized treatment planning.

At the early stage of this study, we first evaluated the predictive value of routine clinical indicators for EGFR mutation status. Among them variables, NSE, PT, and TT consistently demonstrated independent contributions in both univariate and multivariate analyses, supporting previous reports that suggest a potential interaction between coagulation processes and the EGFR signaling pathway (de Almeida et al., 2018). In contrast, sex and smoking history were not significantly associated with EGFR mutation status in our cohort, despite the widely reported observation that EGFR mutations are more common in females and never-smokers. Previous studies have reported inconsistent findings regarding the relationship between smoking and EGFR mutations, some suggested an association with rare EGFR mutation subtypes (Maezawa et al., 2024), whereas others found no direct correlation (Ruano-Ravina et al., 2016).

This discrepancy may be attributable to the specific features of our study population, which exclusively includes patients with stage IA NSCLC, many of whom had very small tumors, with 73.9% (Reference Table S1) of lesions presenting as pure or mixed ground-glass opacities. Similar findings have been reported in patients with stage I pulmonary ground-glass opacity lesions (Tang et al., 2025). Consistently, sex and smoking history were not found to be significant predictors of EGFR mutation status (Qu et al., 2025). These observations suggest that, in very early-stage lung adenocarcinoma, the influence of sex and smoking may be less pronounced. The biological effects of smoking may not yet be fully manifested in these small, indolent lesions, or their impact may be masked by other endogenous factors, such as early oncogenic driver alterations. Further studies are warranted to clarify the mechanisms underlying these findings.

After feature selection, smoking history remained among the key variables, indicating that machine learning methods can identify clinical features with potential associations to EGFR mutations. Other selected features included coagulation indicators such as PT and TT. Many research findings and clinical observations suggest that a hypercoagulable state is commonly present in patients with malignancies, particularly lung cancer. However, the relationship between EGFR mutations and venous thromboembolism (VTE), as well as the underlying molecular mechanisms, remains inconclusive. Conflicting results have been reported: for instance, a single-center study by Wang et al. (2019) involving 323 postoperative lung cancer patients suggested that EGFR mutations may be associated with VTE development, whereas Corrales-Rodriguez et al. (2014) reported no significant association between EGFR mutations and the occurrence or progression of VTE.

To date, there are no studies that directly address the relationship between EGFR mutations and specific coagulation parameters such as PT and TT. Nevertheless, abnormalities in PT and TT may indirectly influence tumor progression by altering the tumor microenvironment, promoting thrombosis, or affecting therapeutic responses. Our findings suggest a potential association between EGFR mutations and elevated PT levels along with decreased TT values. however, the complex interplay between EGFR signaling and coagulation function requires further investigation.

Moreover, clinical features are inherently “linear and low-dimensional” making them insufficient to capture spatial tumor heterogeneity. To address this limitation, we incorporated high-resolution CT imaging and constructed high order radiomic features. These included Gaussian–Laplacian filter derived features, multiscale wavelet components, and texture complexity descriptors, which are fully activated within a nonlinear analytical framework. Compared with traditional statistical models, these CT-based features can capture subtle patterns, such as gray-level run lengths, regional entropy, and long-range dependencies, that are imperceptible to the human eye, thereby providing an additional nonlinear dimension for predicting EGFR mutation status.

In recent years, studies have reported that machine learning models based on CT texture features achieved an AUC of approximately 0.60 in independent validation cohorts (Hao et al., 2024; Deng et al., 2025; Liu et al., 2016; Mei et al., 2018). However, these models were largely limited to first-order statistical features and did not incorporate clinical variables. In our search for the optimal predictive model, LR achieved the best performance, consistent with previous studies. In the classification of EGFR mutation status, LR demonstrated notable advantages and strong interpretability due to its linear decision boundary and explicit feature weighting. By combining LASSO and RFE, LR reduced the high dimensional feature space to 30 key variables. This process effectively removing redundant information while preserving radiomic features and clinically relevant coagulation inflammatory markers associated with EGFR mutation mechanisms. With a test set AUC of 0.745, the model maintained strong performance while minimizing the risk of overfitting.

The model coefficients directly reflect the contribution of each variable, allowing clinicians to better understand which features drive the prediction of EGFR mutation statues, Although some studies have incorporated clinical variables, they often relied solely on data-driven modeling without systematically evaluating the impact of feature selection strategies on predictive performance (Kim et al., 2024). Our results suggest that the sigmoid output of LR is naturally suited to binary classification tasks, and its convex loss function enables rapid convergence even in small-sample, high-dimensional, and sparse settings. Several clinical variables, particularly inflammation- and coagulation-related markers, showed meaningful associations with radiomic features, indicating that these variables may capture complementary biological information relevant to EGFR mutation status.

Overall, this pipeline demonstrates the synergistic value of integrating high order radiomic features with clinical variables and provides a non-invasive, interpretable approach for EGFR mutation screening in stage IA NSCLC. The proposed model achieved stable predictive performance and highlights the complementary contribution of routinely available clinical indicators, particularly inflammation- and coagulation-related markers, to CT radiomic features. However, this study was retrospective and based on data from a limited number of institutions, which may introduce selection bias and limit the generalizability of the findings. Future studies incorporating larger and more diverse multicenter cohorts, along with advanced modeling approaches such as deep learning, may further improve predictive performance and model generalizability, providing additional support for the application of non-invasive EGFR mutation prediction in precision medicine.

## Conclusions

This study included a cohort of 375 patients, from whom clinical data, slice chest CT images, and EGFR mutation status were collected. Radiomic features were extracted from CT images after rigorous manual segmentation of VOIs and consistency validation. A three-stage feature selection strategy was employed, incorporating multiple combinations of selection methods. The optimal feature subset was obtained through an initial variance threshold filter, followed by LASSO regression for dimensionality reduction, and finally refined using RFE, resulting in a set of 30 core features with the highest predictive contribution.

EGFR mutations in this cohort were predominantly exon 21 mutation (L858R, 45.6%) and exon 19 deletions (EX19Del, 34.1%), together accounting for approximately 80% of all mutations. The analysis revealed weak correlations between radiomic features and clinical variables, suggesting that clinical indicators can serve as complementary inputs to enhance the predictive accuracy of downstream tasks. However, certain clinical features such as body weight, BMI, sex, and Fib showed moderate correlations with imaging features. Among the clinical variables, CEA, NLR, PT, and TT were assigned higher weights in both the Lasso-LR and Logistics-LR models, indicating a strong relationship between coagulation-tumor pathways and EGFR mutation status. The most informative CT-derived features were primarily related to Gaussian–Laplacian filtered texture descriptors, highlighting the significance of gray-level inhomogeneity in identifying molecular subtypes.

Further analysis of the selected features indicated that smoking history, PT, and TT were particularly informative for EGFR mutation prediction. Performance comparisons across seven machine learning algorithms (LR, DT, RF, MLP, SVC, NB, and KNN) revealed that each had distinct strengths and weaknesses. LR outperformed others in terms of predictive accuracy, achieving an AUC of 0.74.

In clinical settings where molecular testing is limited or inaccessible, this multimodal predictive model offers a non-invasive, low-cost, and efficient alternative to estimate EGFR mutation status, thereby supporting timely initiation of EGFR-targeted therapies and facilitating personalized treatment strategies.

## Supplemental Information

10.7717/peerj.21579/supp-1 Supplemental Information 1 Code.

10.7717/peerj.21579/supp-2 Supplemental Information 2 Raw Data.

10.7717/peerj.21579/supp-3 Supplemental Information 3 Distribution of CT nodule types according to EGFR mutation status. Values represent the proportion of patients with pure ground-glass nodules (Pure), part-solid nodules (mix), and solid nodules (solid) in the EGFR-negative, EGFR-positive, and combined cohorts (Negative + Positive). Proportions were calculated within each EGFR group, and the values in each row sum to 1.000. This table illustrates the distribution of nodule appearance patterns on CT across different EGFR mutation groups.

## Additional Information and Declarations

### Data Availability

The following information was supplied regarding data availability:

The raw data and code are available in the Supplemental Files.
