# Supplementary material for: Radiomic and clinical predictors of epidermal growth factor receptor mutation in stage IA non-small cell lung cancer
Source: PeerJ. 2026 Aug 3;14:e21579. doi: 10.7717/peerj.21579 (PMC13446228; doi:10.7717/peerj.21579)
Supplement: Supplementary file 1 [file peerj-14-21579-s003.docx]

Supplement Table 1. Distribution of CT nodule types according to EGFR mutation status

|  | **Pure** | **mix** | **solid** |
| --- | --- | --- | --- |
| Negative | 0.244 | 0.445 | 0.311 |
| Positive | 0.099 | 0.678 | 0.223 |
| Negative+Positive | 0.163 | 0.576 | 0.261 |
